# Genome-wide polygenic scores for age at onset of alcohol dependence and association with alcohol-related measures

**DOI:** 10.1038/tp.2016.27

**Published:** 2016-03-22

**Authors:** M Kapoor, Y-L Chou, H J Edenberg, T Foroud, N G Martin, P A F Madden, J C Wang, S Bertelsen, L Wetherill, A Brooks, G Chan, V Hesselbrock, S Kuperman, S E Medland, G Montgomery, J Tischfield, J B Whitfield, L J Bierut, A C Heath, K K Bucholz, A M Goate, A Agrawal

**Affiliations:** 1 Neuroscience Genetics & Genomics Department of Neuroscience, Icahn School of Medicine at Mount Sinai, New York, NY, USA; 2 Washington University School of Medicine, St. Louis, MO, USA; 3 Indiana University School of Medicine, Indianapolis, IN, USA; 4 Queensland Institute of Medical Research, Brisbane, QLD, Australia; 5 Rutgers University, New Brunswick, NJ, USA; 6 University of Connecticut Health Center, Farmington, CT, USA; 7 University of Iowa Carver College of Medicine, Iowa City, IA, USA

## Abstract

Age at onset of alcohol dependence (AO-AD) is a defining feature of multiple drinking typologies. AO-AD is heritable and likely shares genetic liability with other aspects of alcohol consumption. We examine whether polygenic variation in AO-AD, based on a genome-wide association study (GWAS), was associated with AO-AD and other aspects of alcohol consumption in two independent samples. Genetic risk scores (GRS) were created based on AO-AD GWAS results from a discovery sample of 1788 regular drinkers from extended pedigrees from the Collaborative Study of the Genetics of Alcoholism (COGA). GRS were used to predict AO-AD, AD and Alcohol dependence symptom count (AD-SX), age at onset of intoxication (AO-I), as well as maxdrinks in regular drinking participants from two independent samples—the Study of Addictions: Genes and Environment (SAGE; *n=*2336) and an Australian sample (OZ-ALC; *n=*5816). GRS for AO-AD from COGA explained a modest but significant proportion of the variance in all alcohol-related phenotypes in SAGE. Despite including effect sizes associated with large numbers of single nucleotide polymorphisms (SNPs; >110 000), GRS explained, at most, 0.7% of the variance in these alcohol measures in this independent sample. In OZ-ALC, significant but even more modest associations were noted with variance estimates ranging from 0.03 to 0.16%. In conclusion, there is modest evidence that genetic variation in AO-AD is associated with liability to other aspects of alcohol involvement.

## Introduction

Multiple epidemiological and genetically informed studies have documented the importance of age at onset of alcohol dependence (AO-AD) as a key feature of sub-types of alcoholism that vary in etiology and severity.^1, 2, 3, 4, 5, 6^ For instance, Cloninger *et al.*^3^ identified Type I and II alcoholics who were distinguished by, among other features, age at onset of alcohol problems. Similarly, Babor *et al.*^2^ defined Type A and B alcoholics—the latter were distinguished by early onset of alcohol problems. Across these typologies, early-onset problematic use was consistently associated with a more severe form of the disorder, which was often accompanied by polysubstance use and other psychiatric comorbidity, particularly externalizing disorders.

AO-AD is also correlated with other features of drinking. For instance, earlier AO-AD is associated with more alcohol dependence symptoms,^2^ and this relationship may be influenced by shared genetic liability. Cloninger *et al.*^7^ also posited that Type II/early-onset alcoholism may represent a more heritable form of the disorder. While there have not been studies that examine the heritability of AO-AD, numerous studies have robustly documented the role of additive genetic influences on AD itself^8, 9^ and on both age at first drink^10, 11, 12^ and the speed of transitioning from first use to the development of alcohol problems.^13, 14^ In support of these findings, a recent genome-wide association study (GWAS) in extended pedigrees from the Collaborative Study of the Genetics of Alcoholism (COGA) found several single nucleotide polymorphisms (SNPs) that were significantly associated with AO-AD (*P<*5E−8).^15^ For rs2168784, the most significant SNP, 30% of those homozygous for the minor allele met criteria for alcohol dependence while only 19% of those homozygous for the major allele did. This SNP was also associated with AD diagnosis in the COGA dataset.

To our knowledge, none of the top SNP effects identified in the previous study by Kapoor *et al.*^15^ have yet been replicated. However, the significance associated with individual top SNPs might be subject to sample-specific characteristics (for example, families densely affected for alcoholism). Recently, investigators have begun to use genome-wide risk scores (GRS) that reflect the polygenic and aggregate nature of genotypic effects. Effect sizes generated for one phenotype in a given sample can be used to generate GRS in additional samples; the association between these GRS and a similar phenotype may be seen as evidence for replication while correlations between the GRS and other related phenotypes provide support for shared genetic underpinnings.^16^

The present study utilizes GRS generated from the analysis of AO-AD in COGA (discovery sample) conducted by Kapoor *et al.*^15^ and applies these scores to two independent samples, the Study of Addictions Genes and Environment (SAGE; using the portion independent of the COGA subjects who were in the discovery sample)^17^ and a sample of Australian subjects (OZ-ALC).^18^ In contrast to COGA, which is comprised of extended pedigrees with a high rates of alcohol dependence, SAGE consisted of alcohol-dependent cases and alcohol exposed but non-dependent controls. Even though SAGE subjects were drawn from studies that used family history of alcohol and drug dependence to ascertain cases, including COGA, all overlapping subjects were removed and all subjects were unrelated to each other. OZ-ALC, on the other hand, consisted of pedigrees that were derived from various sources, including family studies ascertained for heavy drinking and heavy smoking and a sample consisting of large sibships. Despite being similar to COGA for sibship size, the density of alcohol-related problems in the OZ-ALC pedigrees is substantially lower. The variability in SAGE and OZ-ALC allowed us to investigate the generalizability of the COGA findings.

Overall, we were interested in replicating and generalizing our prior findings and extending them to other alcohol-related phenotypes. Thus, our goals were twofold: first, we examine whether GRS created from the GWAS of AO-AD in the COGA discovery sample^15^ is associated with AO-AD in the independent portion of SAGE and in OZ-ALC. Second, we examine whether GRS for AO-AD is associated with other features of alcohol involvement, including age at first intoxication, lifetime maximum drinks in a 24-h period and number of symptoms and diagnosis of alcohol dependence in the SAGE and OZ-ALC datasets.

## Materials and methods

### Sample

Data were drawn from the three sources described below. The institutional review board at each contributing institution reviewed and approved the protocols.

### Discovery sample

The discovery sample was genome-wide SNP data on 1788 regular drinkers (defined below) from 118 large European-American families densely affected with alcoholism;^15^ subjects from that study who were not regular drinkers were excluded. Ascertainment was based on a proband in treatment for alcohol dependence who had at least two first-degree relatives affected by alcohol dependence. Of these subjects, 685 met criteria for DSM-IV alcohol dependence (mean age of onset 22.5 years). A genome-wide Cox proportional hazards regression model was used to test the association between age at onset of AD and 4 058 415 imputed SNPs with minor allele frequency ⩾5%.^15^ A robust sandwich variance estimators approach was used to account for the familial correlation among observations (https://cran.r-project.org/web/packages/survival/survival.pdf).

### Replication samples

SAGE consisted of 2593 unrelated European-American subjects. Of these, 2336 individuals who reported regular drinking were included in these analyses. Subjects were selected from three large, complementary studies: COGA,^19^ Family Study of Cocaine Dependence (FSCD)^20^ and Collaborative Genetic Study of Nicotine Dependence (COGEND).^21^ Further details of the SAGE sample are available elsewhere.^17^ One hundred and twenty nine individuals who were both in SAGE and the COGA discovery sample were excluded. The sample consisted of alcohol-dependent cases (*N=*1167, mean age at onset 24.7 years) and alcohol-exposed controls (*N=*1169).

The OZ-ALC sample consisted of 6169 individuals (for this study, 5816 regular drinkers were included) from 2356 families ascertained from 3 coordinated studies derived from a larger Australian twin registry: (i) the Nicotine Addiction Genetics (NAG) Study which ascertained heavy smoking index cases; (ii) the OZ-ALC-EDAC study, which ascertained index cases with a history of alcohol dependence or scoring above the 85th centile for heaviness of drinking factor score (operationalized as in the study by Grant *et al.*^22^); (iii) the OZ-ALC-BIGSIB study, which ascertained large sibships (4–14 full siblings), regardless of sibling phenotypes. Further details regarding recruitment may be found in the study by Heath *et al.*^18^ OZ-ALC was not ascertained for alcohol dependence (although some contributing studies were ascertained for heavy smoking and heavy alcohol consumption) and includes 1714 alcohol-dependent individuals (mean age of onset 26.3 years).

### Phenotypic assessments

The discovery and replication samples utilized versions of the Semi-Structured Assessment for the Genetics of Alcoholism (SSAGA)^23, 24^ to obtain interview-based self-report data on ages of onset and other alcohol-related measures. In the discovery sample, AO-AD was defined as the age at which individuals reported first experiencing three or more of seven DSM-IV alcohol dependence criteria clustering within a 12-month period. Only individuals who were regular drinkers (that is, reported a lifetime history of drinking at least once a month for 6 months or longer) were included. As is the norm for Cox survival modeling, those who did not meet criteria for DSM-IV AD were censored at their age at interview.

For the present analyses, in addition to AO-AD, which was coded identically as in the discovery sample, the following measures were drawn from SAGE and OZ-ALC:

### Alcohol-related measures

AO-I, defined as the age at which the respondent reported first getting drunk (that is, their speech was slurred or they felt unsteady on their feet).

AD diagnosis (binary) was based on DSM-IV; individuals who endorsed three or more criteria that clustered within a single 12-month period were diagnosed with AD.

Alcohol dependence symptom count (AD-SX) was defined as the sum of the seven DSM-IV dependence criteria.

Maxdrinks, defined as the maximum number of drinks consumed in a single 24-h period during their lifetime. The measure was Winsorized at the 95th percentile (>100 drinks) and log (10) transformed for analyses.

### Negative control

Height, via self-report, was used as a negative control.

### Genotyping in discovery sample

Genotyping was conducted using the Illumina OmniExpress array (Illumina, San Diego, CA, USA). A total of 4 058 415 SNPs that were imputed in BEAGLE (https://faculty.washington.edu/browning/beagle/beagle.html) were analyzed. Further details are available in the manuscript by Kapoor *et al.*;^15^ data are available at dbGaP phs000763.

### Genotyping in replication samples

For SAGE, DNAs were genotyped on the Illumina Human 1 M beadchip (Illumina) by the Center for Inherited Diseases Research (CIDR) at the Johns Hopkins University; data are available at dbGaP phs000092. A total of 948 658 SNPs passed data-cleaning procedures and further within sample filtering for autosomal and X-chromosome markers yielded 948 142 markers. HapMap genotyping controls, duplicates, related subjects and outliers were removed from the sample set.^17^ The software package EIGENSTRAT^25^ was used to calculate principal components reflecting ancestral differences. Only genotyped SNPs were selected from SAGE, resulting in 669 984 overlapping SNPs which were further pruned for linkage disequilibrium (maximum pairwise *r*^2^=0.25 within sliding windows of 100 SNPs), resulting in 90 365 SNPs that were used for all subsequent analyses.

For OZ-ALC, most subjects (*N=*4601) were genotyped on the Illumina CNV370-Quadv3 (Illumina); genotyping on a small number of additional individuals was conducted on the Illumina 317 K (*N=*20) and 610 Quad v1 (*N=*517) platforms; data are available at dbGaP phs000181 (see the study by Medland *et al.*,^26^ for additional details). To account for the lower density of genotyped SNPs and variation in platform contents, imputation to HapMap (http://hapmap.ncbi.nlm.nih.gov) CEU I+II data (release 22, build 36) was conducted in MACH^27^ and best guess genotypes were selected based on *R*_sq_⩾0.3 and imputation quality⩾0.9, resulting in 112 594 autosomal SNPs. Nuanced admixture was determined using EIGENSTRAT^25^ and outliers were removed, as outlined in the study by Heath *et al.*^18^

### Association using GRS in replication datasets

Based on effect sizes for the analysis of AO-AD generated in the discovery (COGA) sample, GRS at *P*-value thresholds of 0.01 (GRS_0.01_), 0.05 (GRS_0.05_), 0.10 (GRS_0.1_) and 0.50 (GRS_0.5_) were created in SAGE and OZ-ALC sample using PLINK^28^ and SAS (SAS Institute, Cary, NC, USA). Briefly, SNPs in COGA that were significant at each *P*-value threshold (for example, *P<*0.01) were selected. For each SNP, the effect size was calculated as the natural logarithm transformation of the hazard ratio from COGA. For every individual in SAGE and OZ-ALC, this effect size was multiplied by the number of copies of reference allele, and this product was summed across all SNPs.^28^ For the SAGE dataset, the number of SNPs for each score was: GRS_all_ (110 797), GRS_0.5_ (58 374), GRS_0.1_ (12 254), GRS_0.05_ (6147) and GRS_0.01_ (1441), while for OZ-ALC number of SNPs for each score was: GPS_all_ (112 594), GPS_0.5_ (57 053), GPS_0.1_ (12 161), GPS_0.05_ (6268) and GPS_0.01_ (1402). The resulting GRS was used to predict AO-AD, as well as other measures (AO-R, AO-I, AD-SX and Maxdrinks) in those datasets. Associations between age of onset measures and GRS were conducted using Cox proportional hazards analysis. Logistic and linear regression was used for dichotomous (AD) and continuously distributed (AD-SX, Maxdrinks) measures, respectively. Similar to the analysis in the discovery sample, a robust sandwich variance estimator approach was used to account for the familial correlation among OZ-ALC families. For both studies, sex, age at last interview and study source (COGEND vs FSCD vs COGA; NAG vs EDAC vs BIGSIB) were included as covariates.

### Sensitivity analysis

Based on recommendations by Dudbridge,^29^ that replication of polygenic scores is optimized when the size of the discovery and test samples is approximately equal; we performed 10 000 iterations in which we randomly resampled 1788 individuals from the pool of 2336 subjects in SAGE. Cox proportional hazard models were fit to each randomly drawn sample to examine whether the magnitude of the association was modified by selection of a comparatively sized test sample. Similar analyses were not conducted in OZ-ALC as random selection of subsets of individuals nested in pedigrees would not be representative of the sampling design nor would selection of subsets of whole pedigrees allow for adequate numbers of individuals with AO-AD.

## Results

### Sample characteristics

Characteristics of the replication samples, SAGE and OZ-ALC, are presented in Table 1. In both samples, those meeting criteria for AD (*N*_SAGE_=1167; *N*_OZ-ALC_=1714) were more likely to report an earlier AO-I. They also reported higher Maxdrinks. In general, individuals from SAGE were heavier drinkers and have a greater number of AD-SX than those from OZ-ALC. This is unsurprising given the differences in ascertainment strategies. Modestly, earlier onset of drinking to intoxication and AD was noted in SAGE relative to OZ-ALC. No differences in height were noted across individuals with and without AD or across SAGE and OZ-ALC.

### Association between GRS and alcohol measures

**Figure 1:**
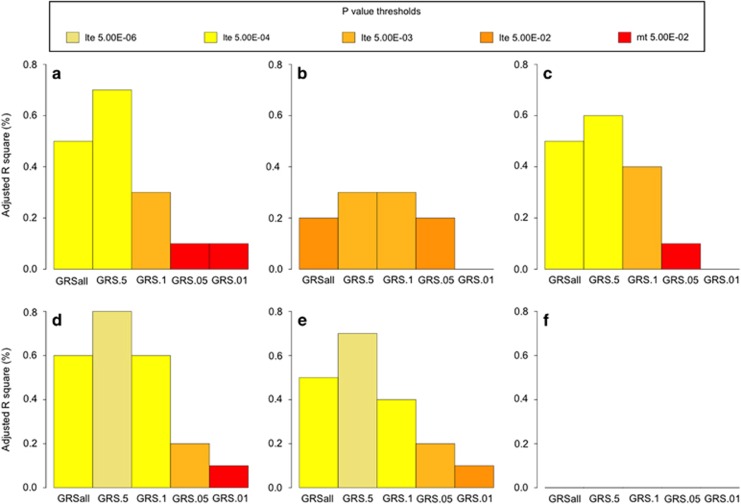
GRS generated from an analysis of AO-AD in a discovery sample, at varying *P*-value thresholds, predicting (**a**) AO-AD, (**b**) AO-I, (**c** ) AD, (**d**) AD-SX, (**e**) Maxdrinks and (**f**) height in SAGE dataset. The *x*-axis represents the GRS thresholds and *y-*axis represents the adjusted *R*^2^ for the trait. Each bar represents the values of adjusted *R*^2^ for SAGE. Colors of the bar represent the level of significance achieved. AD, alcohol dependence; AD-SX, total number of DSM4 AD symptoms endorsed; AO-AD, age at onset of AD; AO-I, age at onset of intoxication; GRS, genome-wide polygenic scores; SAGE, Study of Addictions: Genes and Environment.

As shown in Table 2 and Figure 1, GRS for AO-AD were significantly associated with AO-AD and also with AD in SAGE for cutoffs above GRS_0.05_. Increasing *P*-value thresholds resulted in greater proportion of variance explained with the adjusted *R*^2^ ranging from 0.3% for GRS_0.1_ to 0.7% for GRS_0.5_. The variance explained was maximum when we included the SNPs with *P*⩽0.5. In addition to AO-AD, GRS explained a modest proportion of the variance in AO-I (≈0.3%). GRS from COGA were also related to AD-SX and Maxdrinks in SAGE, explaining 0.2 to 0.8% of the variance in these measures.

**Figure 2:**
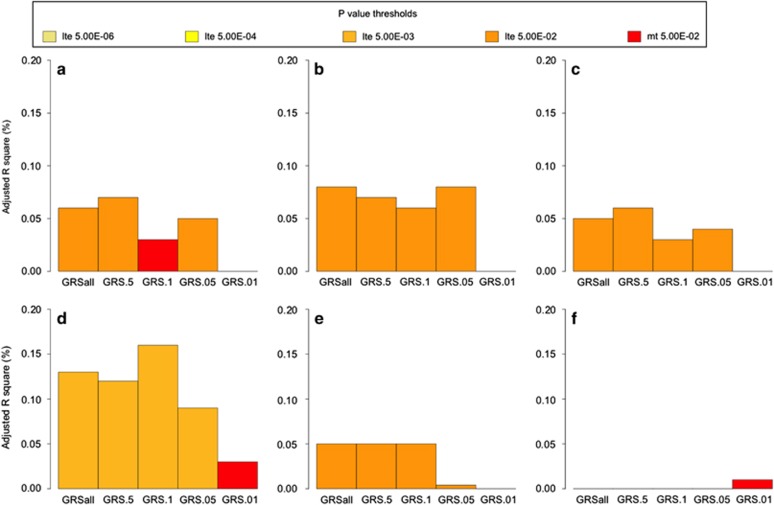
GRS generated from an analysis of AO-AD in a discovery sample, at varying *P*-value thresholds, predicting (**a**) AO-AD, (**b**) AO-I, (**c** ) AD, (**d**) AD-SX, (**e**) Maxdrinks and (**f**) height in OZ-ALC dataset. The *x*-axis represents the GRS thresholds and *y*-axis represents the adjusted *R*^2^ for the trait. Each bar represents the values of adjusted *R*^2^ for OZ-ALC. Colors of the bar represent the significance level achieved. AD, alcohol dependence; AD-SX, total number of DSM4 AD symptoms endorsed; AO-AD, age at onset of AD; AO-I, age at onset of intoxication; GRS, genome-wide polygenic scores.

In contrast, AO-AD GRS from COGA explained only a very modest (but significant) proportion of variation in AO-AD (0.06–0.07%) and AD (0.03–-0.06%) in OZ-ALC (Table 2 and Figure 2). The GRS were modestly associated with AO-I (0.2–0.3%), as well as with AD-SX (0.09–0.13%) and Maxdrinks (0.004–0.05%) in OZ-ALC. The associations were far less significant than those noted in SAGE, explaining ⩽0.16% of the variance in alcohol-related phenotypes. Height was included as a negative control and was not associated (*P>*0.05) with any GRS across SAGE and OZ-ALC.

Resampling 10 000 subsets of SAGE individuals to create a sample size that was equivalent to COGA resulted in similar results. GRS showed statistically significant association with the AO-AD (*P<*0.05) in the reduced SAGE dataset in all 10 000 permutations. However, the reduction in sample size influenced the magnitude of *P*-values and only about 12% of the time, the association *P*-values were equal to or more significant than the original *P*=6.70 × 10^−06^ observed with the full SAGE sample.

## Discussion

Using effect sizes generated via a prior GWAS of AO-AD in the COGA family sample,^15^ we created GRS at varying *P*-value thresholds and examined their association with AO-AD, AD, AO-I, as well as liability to problematic drinking (AD-SX and Maxdrinks) in two independent and differently structured and ascertained datasets, SAGE and OZ-ALC. GRS, especially when including SNPs associated with AO-AD at more liberal *P*-value thresholds, were significantly associated with a range of these alcohol-related measures in those two independent and distinctly ascertained samples. In contrast, there was no evidence for replication of the top 10 most significantly associated variants from COGA in either SAGE (6.6 × 10^−1^–8.1 × 10^−1^) or OZ-ALC (8.7 × 10^−1^–8.9 × 10^−1^). This strongly underscores the idea that multiple common genetic effects contribute to the etiology of complex disorders like addictions.

GRS comprised of >110 000 SNPs only captured very modest proportions of the variance in any alcohol-related measure (<1%). This observation is consistent with other studies.^30, 31, 32^ For instance, Vink *et al.*^32^ used GRS constructed from a large meta-analysis of GWAS of tobacco smoking measures to predict variance in alcohol, tobacco and cannabis-related outcomes. In that study, polygenic scores that were associated with tobacco-related measures at *P<*10^−70^ explained, at most, 1.5% of the variance in any substance-related outcome. Similarly, Power *et al.*^30^ examined the relationship between cannabis involvement and GRS generated from a meta-analysis of schizophrenia (*N=*13 833 cases, 18 310 controls) which included 13 genome-wide significant loci. Even though schizophrenia GRS were significantly associated with cannabis use, the scores, even at *P<*0.05 explained <1% of the variance in cannabis-related phenotypes. For alcohol-related measures, Salvatore *et al.*^31^ found that GRS generated for alcohol problems (*N=*4304) only predicted 0.6% of the variance in a similar measure in an independent sample. Despite relying on a smaller discovery sample (COGA, *N=*1788), our findings are consistent with these estimates. Nonetheless, the small sample size of the discovery set limits the accuracy of predicted SNP effect sizes and likely influenced our ability to generate GRS that might be reliable predictors of alcohol involvement in independent samples.

An additional consideration when viewing these results is the difference in ascertainment method across COGA, SAGE and OZ-ALC. The discovery sample (COGA) consisted of extended pedigrees ascertained for a dense family history of alcoholism and it was not expected that all variants associated with alcohol-related measures in such densely affected pedigrees would generalize to other cohorts. SAGE cases were selected for DSM-IV alcohol dependence from among several studies focused on alcohol, tobacco and cocaine and thus, as expected, we note a stronger degree of replication in this sample. In contrast, OZ-ALC comprises of samples ascertained for heavy smoking, discordance of heavy alcohol consumption measures and also for large sibship size (without any oversampling for substance-related phenotypes). Therefore, it is not surprising that replication in OZ-ALC, a less severely affected sample, is weaker for AO-AD, but occurs for ages of onset for earlier drinking milestones (for example, AO-I) and for measures that are quantitative indices of problem drinking (for example, AD-SX). In OZ-ALC, AO-I and AO-R, as well as AD-SX and Maxdrinks may serve as proxies for genetic liability to problematic drinking, while in COGA and SAGE this liability may be appropriately captured by AO-AD itself. Even so, the statistical significance of the associations and the proportions of variance explained in OZ-ALC are markedly lower than those in SAGE. Nonetheless, as OZ-ALC is so markedly distinct from COGA and SAGE, any level of association between COGA GRS and alcohol-related measures in OZ-ALC may be considered as support for the generalizability of the COGA results.

Dudbridge *et al.*^29^ has noted that there are two purposes for GRS: association testing (that is, replication, reliant on significance/*P*-value) and prediction of phenotypic variance (for example, reliant on *R*^2^ estimates). Based on numerous simulations, he concluded that while most studies with approximately equally sized training (that is, COGA) and testing (for example, SAGE) samples are well-powered for association testing, current training samples are underpowered for prediction. Consistent with this observation, we were interested in the former rather than the latter and even though we report *R*^2^ values, the emphasis of our analysis was association testing. We confirm that selecting a testing sample of approximately the same size as the training cohort, as was achieved via our bootstrapping approach, yields significant association. Dudbridge^29^ notes that 10-fold cross-validation might be a more efficient approach to maximizing prediction. As two of our samples are family-based (COGA and OZ-ALC), such cross-validation approaches, which may necessitate disaggregating members of pedigrees or selecting subsets of pedigrees that may or may not be informative for the etiology of genetically transmitted AD, may not be applicable. Hence, our findings should be observed in the context of association (and not prediction) testing alone.

Finally, it is worth noting that we did not attempt to characterize the SNPs that comprised each GRS nor did we create biologically informed GRS by selecting variants that related to a specific neurotransmitter pathway (for example, dopamine variant profile). Given the high false discovery rates typically associated with ascribing a functional direction to such variants of purported biological importance (except, perhaps, the alcohol dehydrogenase variants) and our currently limited understanding of the etiology of AD, we employed a more conservative and agnostic approach of utilizing genome-wide data. Future studies that contrast such genome-wide PRS with biologically informed risk scores may be valuable in the construction of the architecture underlying the polygenicity associated with complex traits such as AO-AD. Nonetheless, our approach precludes any mechanistic interpretation of the polygenicity represented by each GRS.

In conclusion, using GRS generated for AO-AD, we document that similar genetic factors might underpin the liability to alcohol dependence, drinking to intoxication and indices of problematic drinking in independent datasets. Future studies could strengthen these interpretations by conducting meta-analyses across multiple samples to produce larger discovery samples for generating GRS that could be applied to a wider range of alcohol and other substance phenotypes.

## Figures and Tables

**Table 1:** Characteristics of 2593 European-American subjects, stratified by diagnosis of DSM-IV AD in the regular alcohol drinkers in the replication samples of SAGE and OZ-ALC.

	*SAGE*			*OZ-ALC*		
	*All regular drinkers (*N=*2336)*	*Alcohol dependent (*N=*1167)*	*Not alcohol dependent (*N=*1169)*	*All regular drinkers (*N=*5816)*	*Alcohol dependent (*N=*1714)*	*Not alcohol dependent (*N=*4102)*
Males (%)	1089 (46.6)	710 (60.8)	379 (32.4)	2825 (48.6)	1074 (62.7)	1751 (42.7)
Age at Interview (mean±sd)	38.2±9.5	38.2±9.9	38.3±9.1	44.3±9.3	41.1±8.1	45.61±9.4
						
*Alcohol-related measures*						
Ever got drunk (%)	2274 (97.4)	1167 (100)	1107 (94.7)	5411 (98.9)	1708 (99.7)	3703 (98.35)
AO-I (mean±sd)	17.2±6.6	15.2±3.5^a^	19.3±8.1^a^	18.3±5.6	16.4±2.9^a^	19.1±6.3^a^
Maxdrinks	20.9±19.2	30.3±20.7^a^	11.5 ±11.5^a^	18.4±14.1	26.8±15.5^a^	14.9±11.8^a^
AD-SX	3.1±2.5	5.3±1.5^a^	0.9±0.9^a^	1.9±1.7	4.0±1.2^a^	1.0±1.0^a^
AO-AD (mean±sd)	—	24.7±7.7	—	—	26.3±8.6	—
						
*Control measure*						
Height (in)	67.6+3.9	68.4+3.8^a^	66.9+3.8^a^	67.5±3.9	68.4±3.8^a^	67.1±3.9^a^

Abbreviations: AD, alcohol dependence; AD-SX, total number of DSM4 AD symptoms endorsed; AO-AD, age at onset of AD; AO-I, age at onset of intoxication; DSM-IV, Diagnostic and Statistical Manual IV; Maxdrinks, maximum number of alcoholic drinks in 24 h; SAGE, Study of Addictions: Genes and Environment .

a Student *t*-test, *P<*0.0001.

**Table 2:** GRS generated from an analysis of AO-AD in a discovery sample, at varying *P*-value thresholds, predicting AO-AD, other features of drinking in regular drinkers from SAGE (*N=*2336) and OZ-ALC (*N=*5816)

*Age of onset measures*									*Binary measure*			
	*AO-AD*				*AO-I*				*AD*			
	*SAGE*		*OZ-ALC*		*SAGE*		*OZ-ALC*		*SAGE*		*OZ-ALC*	
	*P*-value	*Adj.* R^2^ *(%)*	*P*-value	*Adj.* R^2^ *(%)*	*P*-value	*Adj.* R^2^ *(%)*	*P*-value	*Adj.* R^2^ *(%)*	*P*-value	*Adj.* R^2^ *(%)*	*P*-value	*Adj.* R^2^ *(%)*
GRS_all_	9.90E−05	0.5	4.23E−02	0.06	1.33E−02	0.2	2.14E−02	0.08	7.98E−05	0.5	2.69E−02	0.05
GRS_0.5_	6.70E−06	0.7	3.03E−02	0.07	6.42E−03	0.3	2.60E−02	0.07	5.26E−06	0.6	2.03E−02	0.06
GRS_0.1_	1.95E−03	0.3	1.09E−01	0.03	4.71E−03	0.3	2.87E−02	0.06	6.88E−04	0.4	4.88E−02	0.03
GRS_0.05_	5.96E−02	0.1	4.88E−02	0.05	2.58E−02	0.2	1.17E−02	0.08	6.96E−02	0.1	4.82E−02	0.04
GRS_0.01_	2.96E−01	0.1	8.52E−01	0.00	3.70E−01	0	1.86E−01	0.00	2.96E−01	0	6.52E−01	0.00

	*AD-SX*				*LOG_10_ Maxdrinks (Maxdrinks)*				*Height (negative control)*			
	*SAGE*		*OZ-ALC*		*SAGE*		*OZ-ALC*		*SAGE*		*OZ-ALC*	
	*P*-value	*Adj.* R^2^ *(%)*	*P*-value	*Adj.* R^2^ *(%)*	*P*-value	*Adj.* R^2^ *(%)*	*P*-value	*Adj.* R^2^ *(%)*	*P*-value	*Adj.* R^2^ *(%)*	*P*-value	*Adj.* R^2^ *(%)*
*Quantitative measures*												
GRS_all_	3.54E−05	0.6	2.41E−03	0.13	7.97E−05	0.5	2.52E−02	0.05	9.54E−02	0	7.93E−01	0.00
GRS_0.5_	2.07E−06	0.8	2.51E−03	0.12	9.56E−07	0.7	1.59E−02	0.05	1.09E−01	0	8.53E−01	0.00
GRS_0.1_	6.40E−05	0.6	1.93E−03	0.16	1.13E−04	0.4	1.38E−02	0.05	9.92E−01	0	4.68E−01	0.00
GRS_0.05_	6.53E−03	0.2	3.59E−03	0.09	5.13E−03	0.2	3.77E−02	0.004	8.49E−01	0	9.43E−01	0.00
GRS_0.01_	6.95E−02	0.1	1.06E−01	0.03	2.58E−02	0.1	8.90E−01	0.00	5.18E−01	0	9.68E−01	0.01

Abbreviations: AD, alcohol dependence; AD-SX, total number of DSM4 AD symptoms endorsed; AO-AD, age at onset of AD; AO-I, age at onset of intoxication; GRS, genome-wide polygenic scores; SAGE, Study of Addictions: Genes and Environment; SNP, single nucleotide polymorphism.

For SAGE, numbers of SNPs for each score were as follows: GRS_all_ (110 797), GRS_0.5_ (58 374), GRS_0.1_ (12 254), GRS_0.05_ (6147) and GRS_0.01_ (1441).

For OZ-ALC: GPS_all_ (112 594), GPS_0.5_ (57 053), GPS_0.1_ (12 161), GPS_0.05_ (6268) and GPS_0.01_ (1402).

The analysis was rerun after removing the MHC region (chr6:28 477 796–33 448 353). There was no change in the adjusted *R*^2^, while *P*-values fluctuated slightly due to small change in number of SNPs.
